# Determining the Quality of Functional Evidence for Coding Variants in *CYP1B1* for Use in Variant Classification

**DOI:** 10.1155/humu/6767909

**Published:** 2026-11-08

**Authors:** Kathryn P. Burdon, Johanna Hadler, Patricia Graham, Stuart W. Tompson, Francesca Pasutto, Kristina Whisenhunt, Owen Siggs, Subhabrata Chakrabarti, Terri L. Young, David A. Mackey, Andrew Dubowsky, Emmanuelle Souzeau

**Affiliations:** ^1^ Menzies Institute for Medical Research University of Tasmania Hobart Tasmania Australia utas.edu.au; ^2^ Department of Ophthalmology Flinders Health and Medical Research Institute Flinders University Adelaide South Australia Australia flinders.edu.au; ^3^ Department of Ophthalmology and Visual Sciences University of Wisconsin-Madison Madison Wisconsin USA wisc.edu; ^4^ Institute of Human Genetics Friedrich-Alexander-Universitätsklinikum Erlangen-Nürnberg Erlangen Germany geneticcentre.org; ^5^ Garvan Institute of Medical Research Sydney Australia garvan.org.au; ^6^ Brien Holden Eye Research Centre L. V. Prasad Eye Institute Hyderabad India lvpei.org; ^7^ Centre for Ophthalmology and Vision Sciences Lions Eye Institute University of Western Australia Perth Western Australia Australia uwa.edu.au; ^8^ SA Pathology Adelaide South Australia Australia sapathology.sa.gov.au

## Abstract

Biallelic variants in the CYP1B1 gene are associated with primary congenital glaucoma and some instances of anterior segment dysgenesis and juvenile open‐angle glaucoma. Genetic testing is increasingly used in the diagnosis of these conditions and to inform future family planning decisions. Variant‐specific functional data play a critical role in variant classification during the interpretation of genetic test results. In this study, the ClinGen Glaucoma Variant Curation Expert Panel (VCEP) evaluated published functional assays assessing CYP1B1 protein stability and enzymatic activity to determine which assays meet the recommended standards for use in variant classification, specifically when applying the PS3 or BS3 criteria from the ACMG/AMP variant interpretation guidelines. Following a comprehensive literature review, the panel identified assays that examined protein abundance and stability, as well as enzymatic activity across a range of substrates. The VCEP determined that BS3 (benign functional evidence) should not be applied to *CYP1B1* as variants exhibit substrate‐specific effects. After detailed review, the panel determined that two assays had an adequate number of validation controls and could distinguish between pathogenic and benign variants. The first measured 17*β*‐estradiol activity using a commercially available luminescence kit in yeast microsomes. The second assessed benzo[a]pyrene activity via HPLC detection, also in yeast microsomes. A consensus review of additional assays recommends the inclusion of more clinical controls to either establish a significant OddsPath or to better define thresholds for applying pathogenic evidence. Incorporating validated functional data into the curation of *CYP1B1* variants will enhance the accuracy of variant classification.

## 1. Introduction

Employing genomic testing in the diagnostic process for heterogeneous rare diseases can pinpoint the cause of disease, improve predictions of prognosis and disease course, and, where possible, inform reproductive and therapeutic options. Primary congenital glaucoma (PCG) and anterior segment dysgenesis (ASD) are a group of eye diseases for which genetic testing is increasingly being used to confirm the diagnosis. This group of disorders has a heterogeneous genetic etiology, with both phenotypes and genotypes overlapping between these two diagnostic categories.

PCG is a developmental glaucoma with onset in the first 2 years of life. It is characterized by elevated intraocular pressure caused by developmental defects of aqueous humor outflow, resulting in buphthalmos, corneal edema, Haab′s striae, and optic disc cupping. Vision loss caused by PCG is irreversible. ASD is a collection of developmental disorders of the anterior segment of the eye, which often leads to secondary glaucoma in young children. The most common genetic cause of PCG is recessive variants in the *CYP1B1* gene. Over 300 variants have been reported and the gene accounts for 5% of PCG in African populations [1], 7%–15% in Asian populations [2–4], 15%–35% in populations of European ancestry [5–7], 30%–35% in Indian populations [8, 9], and up to 90% in Saudi Arabia [10]. The frequency of CYP1B1 variants is proportionately higher in populations with higher prevalence of disease, due to the high rate of consanguinity or founder effects [11, 12]. Biallelic variants in *CYP1B1* have also been reported as the cause of some instances of ASD [13–15] and juvenile open‐angle glaucoma (JOAG) [16–19].

Although *CYP1B1* accounts for the largest proportion of PCG of any gene, challenges remain in determining which variants are pathogenic and in which combinations. *CYP1B1* encodes cytochrome P450 1B1 protein (CYP1B1), a 543 amino acid cytochrome p450 monooxygenase involved in the oxidative metabolism of various substrates including steroid hormones [20] and retinol and fatty acids [21], as well as a broad range of procarcinogenic xenobiotics [20, 21]. Although the impaired substrate metabolism is hypothesized to play a role in the development of PCG, the specific CYP1B1 substrate relevant to this process remains unknown. The enzyme contains a heme‐binding domain at Residues 460–493, which is critical to its function. Variants that eliminate this domain result in no enzymatic activity [22, 23] and are known to be disease‐causing in homozygous or compound heterozygous states [6, 24, 25]. Knockout of the Cyp1b1 gene in mouse models results in a phenotype consistent with human PCG [26]. Missense variants located in the heme‐binding domain or in other regions of the protein can also be classified as pathogenic; functional evidence shows that many of these variants alter enzymatic activity [22, 27, 28], although this may vary depending on the substrate [27].

The *CYP1B1* gene contains at least four common missense variants that together make several common “wildtype” background haplotypes resulting in different isoforms of CYP1B1 protein. The haplotypes are structured across geographical backgrounds [8]. The minimum set of variants used by all studies to define these haplotypes consists of p.Arg48Gly (rs10012), p.Ala119Ser (rs1056827) in Exon 2, and p.Leu432Val (rs1056836) and p.Asn453Ser (rs1800440) in Exon 3. These four variants are in strong linkage disequilibrium with each other. Several publications [29, 30] include p.Asp449Asp (rs1056837); however, this synonymous variant is unlikely to affect protein activity and the effects of the change at the DNA or RNA level have not been assessed. Aklillu et al. [31] also include variant p.Ala443Gly (rs4989888) in the haplotype. There is functional evidence that the baseline enzymatic activity of each haplotype‐associated isoform varies [29, 32], and this may influence the penetrance of certain disease‐causing variants that are not complete null alleles [7], known as hypomorphic alleles. Certain *CYP1B1* variants have been linked exclusively to a limited range of haplotypes worldwide [8]. Observations of incomplete penetrance of certain variants in some families [7] highlight the ongoing challenges in clinically classifying missense variants in *CYP1B1*.

Modern genomic testing involves careful consideration of the clinical classification of any variant identified in a patient, most commonly by assessing the variant against the ACMG/AMP guidelines for variant classification [33]. The guidelines consist of a series of criteria that weight evidence in either the benign or pathogenic direction, then collate the evidence to arrive at a classification of benign, likely benign, uncertain significance, likely pathogenic, or pathogenic. Evidence of pathogenicity is derived from various sources including population data, familial segregation data, computational predictions, and functional experimental evidence in appropriate model systems. To be considered a valid functional test for this purpose, an assay should address the mechanism of disease, have appropriate positive and negative controls and demonstrate a distinctive difference in a sufficient number of known pathogenic and benign control variants [34].

Clinical Genome Resource (ClinGen, http://www.clinicalgenome.org/) Variant Curation Expert Panels (VCEP) are groups of experts working collaboratively to evaluate the evidence available for the clinical classification of each variant in each gene and to determine gene‐ and phenotype‐specific alterations to the ACMG/AMP criteria to improve variant classification. As part of this process for the *CYP1B1* gene, the Glaucoma VCEP reviewed the literature for functionality of variants in *CYP1B1* and determined which assays meet the criteria in the guidelines and can be used in the clinical classification of variants observed in patients with PCG or ASD.

## 2. Methods

We searched PubMed for papers published prior to December 31, 2024, presenting data assessing the function of coding variants in *CYP1B1* reported in people with PCG, ASD, or JOAG using the search string “CYP1B1 AND glaucoma AND (function OR functional) AND (variant OR mutation).” Abstracts were screened and included for further review if they were in English, not a review article and indicated functional testing of CYP1B1 coding variants. Articles without an abstract were retained for further review. Full texts were reviewed for the same inclusion criteria with the addition of testing a relevant disease mechanism (protein abundance, protein stability, or enzymatic activity). We reviewed the reference list of identified papers but did not identify any additional papers. The outcome of the article selection process is depicted in Figure S1. Following review of these papers, it became clear that we needed to assess the activity of the background haplotypes. Additional manual literature searches were undertaken to identify publications testing the enzymatic of background haplotypes but not meeting the main inclusion criteria. An additional five publications were identified.

We generated a list of all variants with available functional data (Table S1). From this, we used the VCEP *CYP1B1* rules V1 (available at https://clinicalgenome.org/affiliation/50053/) to define a set of control variants with available functional evidence that met criteria for classification as likely pathogenic/pathogenic classifications (LP/P) or likely benign/benign (LB/B) without using criteria PS3 (for pathogenic functional evidence) or BS3 (for benign functional evidence).

From each paper, we extracted the characteristics of the assay including the aim of the assay, the vector and cell‐line used, the product measured and the method of detection, details of technical controls, the background haplotype of the constructs, the variants tested, and the result for each variant. From this information, we determined which reports used the same methods (product, vector, cell type, detection method, and background haplotype) and could therefore be combined aiming to assess control data as described by Brnich et al. [34]. We counted the number of LP/P and LB/B control variants available for each unique assay and calculated the OddsPath using the calculator provided in the supporting information of Brnich et al. [34] (OddsPath > 2.1 = PS3_supporting, >4.3 = PS3_moderate, > 18.7 = PS3_strong). Where OddsPath for pathogenic evidence reached at least supporting level, we evaluated the assay results for the control variants to determine assay thresholds for applying PS3. Where overlap was identified, OddsPath was recalculated using the Brnich et al. calculator allowing for one indeterminate result, but no assays met OddsPath requirements under this scenario. Where more than one variant was indeterminate, the assay was excluded from further consideration based on inability to separate benign from pathogenic controls. An assay was required to meet both OddsPath and threshold criteria to be assessed as suitable for the application of PS3.

We also evaluated the variability in the activity of the “wildtype” constructs depending on the background haplotype used. We considered the four common variants c.142C>G (Arg48Gly) rs10012; c.355G>T (Ala119Ser) rs1056827; c.1294C>G (Leu432Val) rs1056836; and 1358A>G (Asn453Ser) rs1800440 as defining the background haplotypes. Variant Leu432Val is often referred to as Val432Leu, with the common allele varying between populations. In some contexts, we refer to it as Val432Leu so that the allele present in the RAVN haplotype, which is used as the reference in most studies, is listed first in the notation.

## 3. Results

We identified 18 reports testing 56 unique variants observed in PCG patients as well as the four common variants or background haplotypes [7, 20, 22, 23, 27–32, 35–42] (Table S1). Seven of the studies specifically tested the activity of the background haplotypes created by the common variants [20, 23, 29, 31, 32, 39, 40]. Assays were described that measured CYP1B1 protein stability, protein abundance, and enzymatic activity for various substrates: 17*β*‐estradiol, ethoxyresorufin, retinol/retinoids, arachidonic acid, and benzo[a]pyrene (Table 1). All the assays used transient transfection but varied in the use of the vector, the cell type, and the detection method (Table 1). The background haplotype of the vector also varied. Some studies tested different variants only on the RAVN haplotype [22, 27, 28, 32, 38], whereas others created constructs specific for each variant of interest on the observed background haplotype [7, 23, 29, 30, 39].

**Table 1:** Characteristics of assays testing functional effects of variants in *CYP1B1*. The number of control variants combined across studies using the same methodology are given, along with the odds of pathogenicity (OddsPath) calculated using the calculator provided by Brnich et al., the level at which PS3 should be applied and the appropriate threshold for application.

Assays	Product measured	Vector	Cell line	Detection method	Haplotype	References using this method	Meets technical controls?	Number of control variants		OddsPath	PS3 level	Threshold for pathogenic
								LP/P	LB/B			
Protein abundance	CYP1B1 protein level	pcDNA3.1‐myc‐His	HEK‐293T	Western blot with anti‐Myc antibody	RAVN	Campos‐Mollo et al. [22]	Yes	11	3	3.0	PS3_supp	N.D.
						Medina‐Trillo et al. [28]	No replicates					
						Lopez‐Garrido et al. [38]	Yes					
					GSLN	Lopez‐Garrido et al. [7]	Yes	1	0	0.0	None	N/A
		pCWori	*E. coli* DH5a	Western blot with chicken anti‐CYP1B1 IgG	?	Choudhary et al. [36]	No replicates	3	2	2.0	None	N/A
						Jansson et al. [37]						

Protein stability	Protein level (inhibition with cycloheximide)	pcDNA3.1‐myc‐His∗	HEK‐293T	Western blot (8 h) with anti‐Myc antibody	RAVN	Banerjee et al. [27]	Yes	16	3	3.0	PS3_supp	N.D.
						Campos‐Mollo et al. [22]						
						Medina‐Trillo et al. [28]						
						Lopez‐Garrido et al. [38]						
	Protein level (inhibition at 4°C)	pCWori	*E. coli* DH5a	Spectrophotometry	?	Choudhary et al. [36]	No replicates	3	2	2.0	None	N/A
						Jansson et al. [37]						
	Protein level in supernatant aliquots over time	pCWori	*E. coli* JM109	Bradford assay	?	Faiq et al. [42]	Yes	0	0	—	None	N/A

17B‐estradiol activity	Luciferin derivative (CYP450‐GLO kit)	?	HEK‐293T	Luminescence 40 h posttransfection	RAVN	Banerjee et al. [27]	Yes	7	2	2.0	None	N/A
		pcDNA3.1‐HA	HEK‐293T	Luminescence 48 h posttransfection	?	Bagiyeva et al. [35]	Yes	1	0	0.0	None	N/A
		pcDNA3	RPE8319	Luminescence 40 h posttransfection	?	Mookherjee et al. [41]	No negative control	1	1	1.0	None	N/A
		pYeDP60	INVSc1‐HR (yeast)	Luminescence	Observed background	Chavarria‐Soley et al. [29]	Yes	2	3	3.0	PS3_supp	< 20% relative activity compared with background haplotype
						Pasutto et al. [30]						
	4‐OH‐E2 and 2‐OH‐E2	pYeDP60	INVSc1‐HR (yeast)	HPLC	RALN	Aklillu et al. [31]	Yes	0	2	—	None	N/A
		pCWori	*E. coli* DH5a	GC/MS	?	Choudhary et al. [36]	No negative control	3	2	2.0	None	N/A
						Jansson et al. [37]						
		?	*E. coli* DH5a	GC/MS	RAVN	Hanna et al. [32]	No negative control	0	4	—	None	N/A
		pCWori	*E. coli* JM109	HPLC	?	Faiq et al. [42]	No negative control	0	0	—	None	N/A

EROD activity	Resorufin	pcDNA3.1‐myc‐His	HEK‐293 T	Fluorometric assay 13–15 h posttransfection, 2 h after substrate added	RAVN	Campos‐Mollo et al. [22]	Yes	11	3	3.0	PS3_supp	N.D.
						Medina‐Trillo et al. [28]						
						Lopez‐Garrido et al. [38]						
					GLSN	Lopez‐Garrido et al. [7]	Yes	1	0	0.0	None	N/A
		pDHFR	COS‐1	Fluorometric assay 48 h posttransfection, 0, 5, and 10 min after substrate added	Observed background	Jeannot et al. [23]	Yes	1	2	1.0	None	N/A

Retinol activity	Retinoic acid via binding to retinoic acid response element (RARE)	?	HEK‐293T	Luminescence at 36 h	RAVN	Banerjee et al. [27]	Yes	7	2	2.0	None	N/A

Retinoid activity	Retinol and retinoic acid	pCWori	*E. coli* DH5a	GC/MS	?	Choudhary et al. [36]	No negative control	1	2	2.0	None	N/A
			*E. coli* JM109	HPLC	?	Faiq et al. [42]	No negative control	0	0	—	None	N/A

Arachidonic acid metabolism	EET and HETE	pCWori	*E. coli* DH5a	GC/MS	?	Choudhary et al. [36]	No negative control	1	2	2.0	None	N/A
			*E. coli* JM109	HPLC	?	Faiq et al. [42]	No negative control	0	0	—	None	N/A

Benzo[a]pyrene activity	Oxidation products of B[a]P‐7,8‐diol	pYes2	*S. cerevisiae* JL20	HPLC	Observed background	Mammen et al. [39]	Yes	6	4	4.0	PS3_supp	< 20% activity compared with background haplotype

*Note:* “?” = information not provided. “∗” = vector not listed for Banerjee 2016. “—” = unable to calculate OddsPath due to zero controls in a category. N/A = not applicable due to insufficient OddsPath. N.D. = threshold not determined due to overlap between pathogenic and benign control variants.

Abbreviations: EET, eicosatetraenoic acids; EROD, ethoxyresorufin O‐deethylation; GC, gas chromatography; HETE, hydroxyeicosatetraenoic acids; HPLC, high performance liquid chromatography; MS, mass spectroscopy.

We first considered whether the assays addressed a relevant disease mechanism and therefore whether the functional criteria BS3 and PS3 should be included in *CYP1B1* variant classification rule specifications. Little is known about the specific molecular mechanisms of glaucoma development or how CYP1B1 is involved. It is known that *CYP1B1* glaucoma is caused through loss of function, evidenced by a knockout mouse with a similar phenotype [26] and null alleles segregating with disease in human families [6, 24, 25]. Although the VCEP could not determine which substrates are important for *CYP1B1* glaucoma development, we were able to conclude that variants resulting in null alleles or significantly reduced enzymatic activity are key in disease pathogenesis and therefore that assays assessing protein abundance or stability as well as enzymatic activity are relevant. For stability assays, a stable protein can still have reduced or absent enzymatic activity and thus, although an unstable protein can be pathogenic, stability alone does not indicate benign effects. Similarly, a lack of effect against one substrate does not indicate no effect against other substrates. Until the field can define the critical substrates, the Glaucoma VCEP concluded that benign evidence (applied as BS3 in the ACMG/AMP criteria) from cell‐based in vitro assays should not be used in formal variant classification for *CYP1B1*.

Evaluation of the enzymatic activity of the background haplotypes to establish the baseline activity of each isoform of the CYP1B1 protein has given mixed results. Figure 1 shows summary findings from each study. Overall, most of the haplotypes appeared to have broadly similar activities when assessed with most methods; however, there are some key differences. Shimada et al. [20] assessed 19 different procarcinogenic xenobiotics as well as a range of steroid hormones against four different human CYP1B1 haplotypes expressed in *E. coli* membranes. They found small differences between haplotypes, and the most active haplotype depended on the substrate. McLellan et al. [40] and Aklillu et al. [31] both tested activity of CYP1B1 expressed in yeast microsomes with HPLC detection and found similar results of no discernible difference between most haplotypes, except the rare haplotype GSVN only tested by Aklillu et al. [31]. Hanna et al. [32] measured 17*β*‐estradiol activity in *E. coli* membranes and found largely similar activity for most haplotypes but reported that the most common haplotype, RAVN, has the lowest activity, with all other haplotypes having twofold to fourfold higher activity. Conversely, Chavarria‐Soley et al. [29] reported that RAVN has the highest 17*β*‐estradiol activity when measured in yeast microsomes, and this is mirrored by data from Mammen et al. [39] assessing benzo[a]pyrene activity, which is also in yeast microsomes. The inconsistency between studies, methods, and substrates makes it challenging to know how to interpret assays testing variants observed in patients. Therefore, we recommend the conservative approach of testing the variant on the background haplotype on which it was observed to most accurately replicate the situation in the patient.

**Figure 1:**
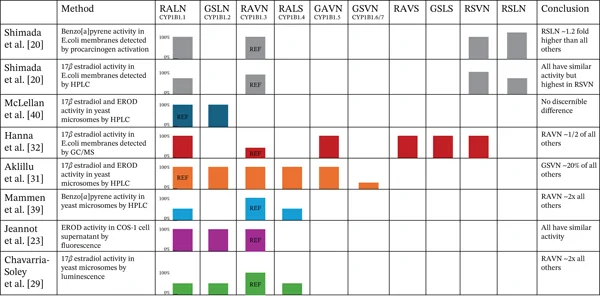
Haplotype comparison results. Image shows cartoons of relative activity of each haplotype tested in each study (not scaled to compare between studies) as well as the method used and the overall conclusion of each paper. Variants included in the haplotype are R48G (Arg48Gly), A119S (Ala119Ser), V432L (Val432Leu), and N453S (Asn453Ser). The haplotype designated by each study as the reference or wildtype is indicated by “REF.” GSVN haplotype is collapsed data from GSVAN and GSVGN with variant A443G (Ala443Gly) included. EROD, ethoxyresorufin O‐deethylation.

Of the variants with functional data in the literature, we identified 13 that were classified as pathogenic, eight as likely pathogenic, one as likely benign, and six as benign using the VCEP *CYP1B1* rules and without the use of PS3 or BS3 (functional data criteria). These 28 variants (Table 2) were used as controls to assess the validity of each assay. Four of the benign controls are the four common variants that make up the background haplotypes: Arg48Gly, Ala119Ser, Val432Leu, and Asn453Ser. Where all components of the assay were the same across multiple papers, we combined papers to count the number of control variants with data available and calculated an OddsPath for each reported assay using the calculator provided by Brnich et al. [34] (Table 1).

**Table 2:** Pathogenic and benign control variants in *CYP1B1* (NM_000104.4) classified with the Glaucoma VCEP modified ACMG/AMP criteria without the use of PS3 or BS3.

Variant	Protein	Rules applied, as per ACMG‐AMP guidelines	Points	Classification	References	ClinVar accession
c.142C>G	p.Arg48Gly∗	BA1, BP4_Mod		Benign	[32, 39]	SCV007106103.1
c.182G>A	p.Gly61Glu	PM3_VS, PP1_Str, PM1, PP3_Mod	16	Pathogenic	[22, 27, 29, 37–39]	SCV007106127.1
c.241T>A	p.Tyr81Asn	BS1, PP3_Mod	−2	Likely benign	[22, 27, 29, 36, 38]	SCV007106128.1
c.317C>A	p.Ala106Asp	PM3_VS, PP1_Mod, PP3_Mod, PM2_Sup	13	Pathogenic	[28]	SCV007106071.1
c.349C>T	p.Arg117Trp	PP1_Str, PM3, PP3, PM2_Sup, PM5_Sup	9	Likely pathogenic	[27, 35]	SCV007106072.1
c.350G>C	p.Arg117Pro	PM3, PM5, PP3, PM2_Sup	6	Likely pathogenic	[27]	SCV007106121.1
c.355G>T	p.Ala119Ser∗	BA1, BP4_Mod		Benign	[32, 39]	SCV007106073.1
c.434_443del	p.Arg145ProfsTer4	PVS1, PM3, PP1_Mod, PM2_Sup	13	Pathogenic	[39]	SCV007106075.1
c.517G>T	p.Glu173Ter	PVS1, PM3_Str, PM2_Sup	13	Pathogenic	[22]	SCV007106076.1
c.685G>A	p.Glu229Lys	BA1		Benign	[22, 23, 27, 29, 36, 38, 41]	SCV007106078.1
c.710C>A	p.Ala237Glu	PM3_Str, PP3, PM2_Sup	6	Likely pathogenic	[28]	SCV007106079.1
c.784G>T	p.Glu262Ter	PVS1, PM2_Sup, PM3_Sup	10	Pathogenic	[22]	SCV007106082.1
c.988_989delinsTT	p.Ala330Phe	PM2_Sup, PM3_Str, PP3, PP1	7	Likely pathogenic	[36]	SCV007670367
c.1033_1035del	p.Leu345del	PM3_Str, PM4, PM1_Sup, PM2_Sup	8	Likely pathogenic	[29]	SCV007106087.1
c.1064_1076del	p.Arg355HisfsTer69	PVS1, PM3_VS, PP1_Str, PM2_Sup	21	Pathogenic	[23]	SCV007106091.1
c.1093G>T	p.Gly365Trp	PS2_Mod, PP3_Mod, PP1, PM1_Sup, PM2_Sup, PM3_Sup	8	Likely pathogenic	[39]	SCV007106094.1
c.1120G>A	p.Asp374Asn	PP1_Str, PM3, PP3, PM2_Sup	8	Likely pathogenic	[39]	SCV007106098.1
c.1159G>A	p.Glu387Lys	PM3_VS, PP1_Str, PP3_Str, PM2_Sup	17	Pathogenic	[7, 27]	SCV007106099.1
c.1168C>A	p.Arg390Ser	PM3_VS, PP1_Str, PP3_Str, PM5, PM2_Sup	18	Pathogenic	[28]	SCV007106100.1
c.1198C>T	p.Pro400Ser	PM3, PP1_M, PP3, PM2_Sup	6	Likely pathogenic	[22]	SCV007106104.1
c.1294C>G	p.Leu432Val∗	BA1, BP4_Mod		Benign	[23, 31, 32, 39]	SCV007106106.1
c.1310C>T	p.Pro437Leu	PM3_VS, PP1_Str, PP3_Mod, PM2_Sup	15	Pathogenic	[28, 39]	SCV007106107.1
c.1328G>C	p.Ala443Gly	BS1, BP4	−5	Likely benign	[30, 38]	SCV007106108.1
c.1331G>A	p.Arg444Gln	PM3_VS, PP1_Str, PP3_Mod, PM2_Sup	15	Pathogenic	[27]	SCV007106109.1
c.1358A>G	p.Asn453Ser∗	BA1, BP4		Benign	[31, 32, 39]	SCV007106111.1
c.1405C>T	p.Arg469Trp	PM3_VS, PP1_Str, PM1, PP3	15	Pathogenic	[22, 27, 37, 39]	SCV007106113.1
c.1409G>A	p.Cys470Tyr	PP3_Str, PM1, PM2_Sup, PM3_Sup	6	Likely pathogenic	[28]	SCV007106115.1
c.1454C>T	p.Ser485Phe	PM3_Str, PM1, PP1_Mod, PP3_Mod, PM2_Sup	11	Pathogenic	[28]	SCV007106116.1
c.1568G>C	p.Arg523Thr	PP1_Str, PP3_Str, PM2_Sup, PM3_Sup	10	Pathogenic	[27, 41]	SCV007106118.1

*Note:* Variants with no points awarded are classified as benign on the basis of standalone criterion BA1. Asterisk “∗” indicates benign variants included in background haplotypes depicted in Figure 1.

Abbreviations: Mod, moderate; Str, strong; Sup, supporting; VS, very strong.

### 3.1. Protein Abundance and Stability

Protein abundance assays were reported in six papers [7, 22, 28, 36–38]. Four of these performed Western blotting of proteins extracted from transfected HEK293T cells using an anti‐Myc antibody 2 h after the substrate for an enzymatic activity assay was added [7, 22, 28, 38]. Three studies used the common RAVN background haplotype [22, 28, 38], whereas the fourth [7] tested the variant on its observed background haplotype (GSLN); therefore, this study is considered separately from the other three when counting control variants tested (Table 1). For the three studies with identical methodology and background haplotype, 11 LP/P controls and 3 LB/B controls were assessed, providing an OddsPath suitable for the application of PS3 at the supporting level (OddsPath = 3.0). However, the studies only presented a qualitative image of a Western blot (Table 3), making it difficult to determine a threshold for indicating a functional effect. The other protein abundance assay reported by two studies [36, 37] used protein expressed in *E. coli* and detected protein levels in a Western blot with chicken anti‐CYP1B1 antibody. These two studies collectively tested three LP/P variants and two LP/B variants, which was insufficient for the application of PS3 at any level (Table 1).

**Table 3:** Data reported for control variants in assays of protein abundance using HEK293T cells and Western blot with anti‐Myc antibody by comparison with wildtype protein.

Variant	Protein	Classification	Campos‐Mollo et al. [22]	Lopez‐Garrido et al. [38]	Medina‐Trillo et al. [28]	Lopez‐Garrido et al. [7]
c.182G>A	p.Gly61Glu	P	Lower	Lower		
c.241T>A	p.Tyr81Asn	LB	Lower	Lower		
c.317C>A	p.Ala106Asp	P			Lower	
c.517G>T	p.Glu173Ter	P	Similar			
c.685G>A	p.Glu229Lys	B	Similar	Similar		
c.710C>A	p.Ala237Glu	LP			Similar	
c.784G>T	p.Glu262Ter	P	Lower			
c.1159G>A	p.Glu387Lys	P				Slightly lower
c.1168C>A	p.Arg390Ser	P			Similar	
c.1198C>T	p.Pro400Ser	LP	Lower			
c.1310C>T	p.Pro437Leu	P			Similar	
c.1405C>T	p.Arg469Trp	P	Lower			
c.1409G>A	p.Cys470Tyr	LP			Similar	
c.1454C>T	p.Ser485Phe	P			Similar	

*Note:* All descriptors are relative to the wildtype construct as defined by each paper (RAVN or GSLN haplotype, see Table 1) at the same time point.

Abbreviations: B, benign; LB, likely benign; LP, likely pathogenic; P, pathogenic.

Protein stability was assessed with three different assays. The first (used in four papers [22, 27, 28, 38]) used cycloheximide to inhibit protein translation of the vector‐derived transcripts in HEK‐293T cells, followed by protein abundance via densitometry of a Western blot targeting a Myc tag on the transfected protein 8 h later. Results for this assay were presented as protein abundance at 8 h expressed as a percentage of 0 h (just prior to cycloheximide addition). The value will be high if the protein is stable (variant has no effect on stability). This assay met an OddsPath of 3.0, suitable for the application of PS3 at the moderate level through the assessment of 16 LP/P and 3 LB/B variants (Figure 2). As shown in Figure 2, the LP/P variants had stability results ranging from 0% (highly unstable) to 100% (highly stable). However, the LB/B control variants were also relatively unstable, making it impossible to recommend a threshold at which this assay could be applied for PS3. Further, the assay was highly variable between reports where the same variant was tested. For example, Arg469Trp was stable when tested by Banerjee et al. [27], but had only 50% protein remaining when measured by Campos‐Mollo et al. [22]. Similarly, the benign variant Glu229Lys had values across the full range in the three studies [22, 27, 38]. In addition, the data presented by Medina‐Trillo et al. [28] indicated that the wildtype protein had only 20% abundance at 8 h (not shown), whereas in the other three papers, the wildtype construct was very stable, with 80% abundance remaining at 8 h. It was not clear why the assay behaved differently for the wildtype construct in Medina‐Trillo et al. nor why specific variants gave such different results when tested in different laboratories. Taking all this together, we do not recommend use of the cycloheximide stability assay for CYP1B1 due to the high variability in the data reported to date.

**Figure 2:**
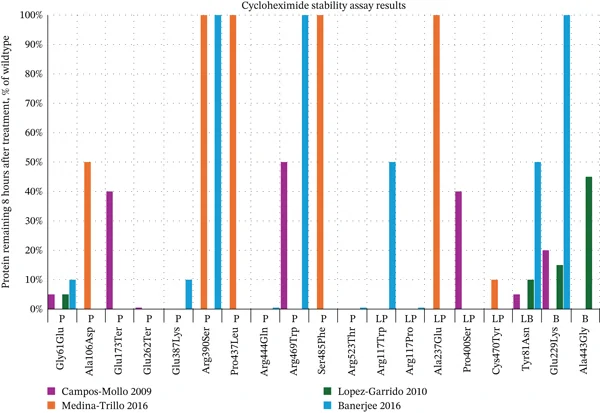
Cycloheximide assay for protein stability on control variants. Results from each study are shown in a different color. The variant classification without the use of functional data is given on the *x*‐axis. Variants are ordered by classification and position in the protein. The percentage of protein remaining after 8 h compared with the RAVN wildtype haplotype is shown. Blank bars indicate that study did not test that variant. Alleles with no detectable activity are indicated by a small bar (0.5%) for the relevant study.

The second stability assay, reported in two papers [36, 37], used *E. coli* cells expressing the protein of interest with translation inhibited by incubation at 4°C. Protein abundance was measured using spectrophotometry. This assay was used to assess three LP/P controls and two LB/B controls, which did not give a sufficient OddsPath to apply PS3 at any level. The third method [42] stored aliquots of the purified protein at various temperatures and assessed the amount of protein remaining in each with a Bradford assay at different time points. No suitable clinical control variants were tested to calculate an OddsPath or apply PS3 for this assay. Taken together, there was insufficient evidence to apply protein abundance or stability data in the classification of variants in *CYP1B1* at this time.

### 3.2. Enzymatic Activity

A variety of different enzymatic activity assays have been reported, testing activity against a range of substrates. 17*β*‐estradiol was the most commonly tested substrate, evaluated in nine studies [27, 29, 30, 32, 35–37, 41, 42] using five different methods (Table 1). Four studies [32, 36, 37, 42] measured estradiol metabolites 4‐OH‐E2 and 2‐OH‐E2 generated from the radiolabeled substrate in membranes of *E. coli* expressing the human CYP1B1 from the pCWori vector. One study used HPLC to detect metabolites [42] and the other three all used gas chromatography mass spectrometry (GC/MS) [32, 36, 37]. Although these three appeared to use the same method, the vector was not given by Hanna et al. [32] and the quoted cloning site did not match that given in the other two studies. Conversely, Hanna et al. [32] did report that all four variants were tested against the RAVN haplotype, which Choudhary et al. [36] and Jansson et al. [37] did not specify. Hanna et al. [32] only tested benign variants, all of which are the defining variants for the background haplotype. None of the studies has tested enough variants to reach a suitable OddsPath individually for PS3.

All other studies evaluating 17*β*‐estradiol activity measured the accumulation of a luciferin derivative with a commercial kit known as the P450 Glo assay system (Promega), but the vector, cell type, and background haplotype varied. Banerjee et al. [27] used HEK293T cells and a construct with the most common RAVN haplotype, but they did not specify the vector. Bagiyeva et al. [35] used the same cell type, with a HA tag in a pcDNA3 vector, but at 48 h instead of 40 h posttransfection. Mookherjee et al. [41] used a pcDNA3 vector in RPE8319 cells, but they did not specify the background haplotype. These differences mean the studies cannot be combined for the purposes of counting control variants and no study tested enough variants individually. Chavarria‐Soley et al. [29] and Pasutto et al. [30] used the pYeDP60 vector to express the protein in microsomes of INVCc1‐HR yeast and expressed each variant on the background haplotype on which it was observed in the patients. This yeast microsome assay was used to test two LP/P variants and three LB/B variants across the two studies (Figure 3) [29, 30], giving an OddsPath of 3.0, suitable for applying PS3 at the supporting level. Both studies measured the molar enzymatic activity and the relative protein abundance, and then calculated the relative activity of each background haplotype and variant by multiplying the two values. They interpreted the variant data by comparison with its specific background haplotype. The activity and abundance for the two relevant background haplotypes investigated in Pasutto et al. were not given in the paper for direct comparison with the variant. However, the background haplotype data were obtained directly from the author (Figure S2), and it was confirmed that they were tested at the same time as the variants. The VCEP concluded that this assay can be used at the supporting level for PS3. By visualization of the relative activity from both papers as the proportion of the background haplotype (Figure 3), suitable thresholds for distinguishing pathogenic from benign variants were able to be determined. We recommend applying PS3 at the supporting level when the variant of interest has a relative activity < 20% compared with the background haplotype.

**Figure 3:**
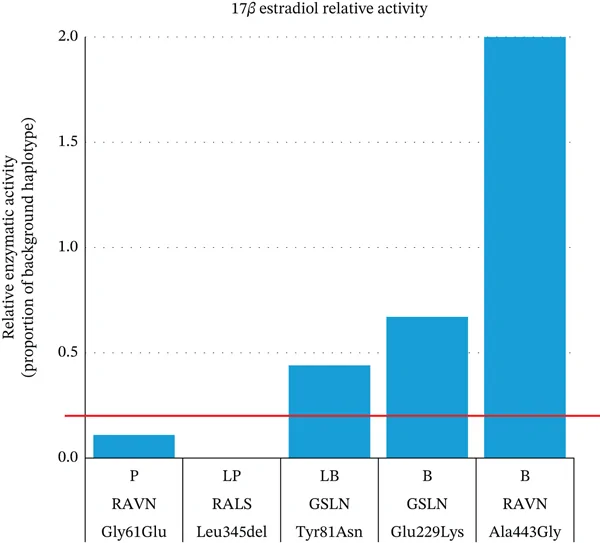
Relative activity against 17*β*‐estradiol of each control variant as a proportion of the background haplotype measured with the yeast microsome assay. Red line represents recommended threshold of 20%, below which PS3_supp can be applied. Ala443Gly data are from Pasutto et al. [30]; all others are from Chavarria‐Soley et al. [29]. The variant classification and the background haplotype used is given on the *x*‐axis.

The ethoxyresorufin O‐deethylation (EROD) assay measured CYP1B1 activity in transfected cells and evaluated the effects of the variant compared with wildtype, which was set at 100% activity. Low values reflected reduced activity compared with wildtype. Four studies [7, 22, 28, 38] measured enzymatic activity in HEK293T cells (Table 1), although Lopez‐Garrido et al. [7] used a different background haplotype, so the assays may not be considered equivalent for control purposes. Jeannot et al. [23] used a similar assay but in a different vector, cell type, and time points (Table 1). Between the three papers using the RAVN haplotype, 11 LP/P variants and 3 LB/B variants were assessed (Figure 4), which gave an OddsPath of 3.0 sufficient to apply PS3 at the supporting level [34]. However, although the LP/P variants all had low activity as expected, the LB/B variants give variable results, overlapping with variants in the LP/P range (Figure 4).

**Figure 4:**
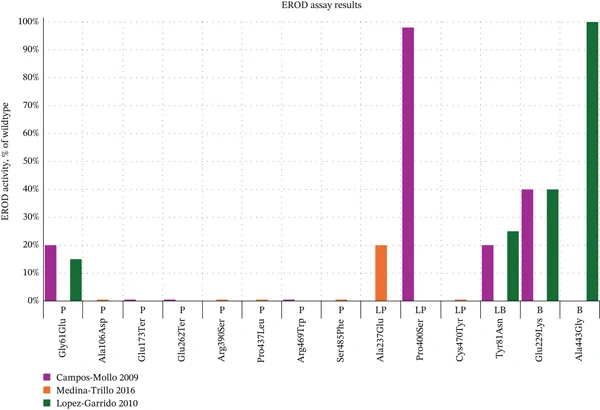
EROD assay for enzymatic activity in control variants. Results from each study are shown in a different color, as a percentage of the activity of the RAVN wildtype construct. The classification of the variant without the use of functional data is given on the *x*‐axis. Variants are ordered by classification and position in the protein. Blank bars indicate that study did not test that variant. Alleles with no detectable activity are indicated by a small bar (0.5%) for the relevant study.

In addition to the protein stability and estradiol activity discussed above, the study of Choudhary et al. [36] also measured activity against retinoids and arachidonic acid, using the same vector (pCWori) and cells (*E. coli*) as for the other assays, with metabolites detected by GC/MS. Only one LP/P control was included and thus there are insufficient controls to apply these assays at any level. Similarly, Faiq et al. [42] used HPLC to measure activity for retinols, arachidonic acid, and melatonin but without the inclusion of appropriate clinical control variants. Retinol activity was measured by Banerjee et al. [27] using a luminescence detection method in HEK293T cells, similar to that applied to estradiol activity. However, the seven LP/P and two LB/B control variants were insufficient to apply PS3 to this assay.

The study by Mammen et al. [39] measured activity against benzol[a]pyrene‐7R‐trans‐7,8‐dihidrodiol. They considered the baseline activity of the background haplotypes (Figure 1) but also tested a range of variants, which have been observed in PCG. The assay was conducted in yeast cells with the oxidation products of the substrate detected with HPLC. The assay assessed six LP/P and four LB/B controls, giving an OddsPath of 4.0, sufficient to apply PS3 at the supporting level (Figure 5). All the benign variants were significantly more active than the RAVN reference haplotype (consistent with data shown in Figure 1) and all the LP/P controls had activity < 12% of the RAVN wildtype (Figure 5). We therefore recommend applying this assay at the supporting level to variants with activity < 20% of the comparator haplotype.

**Figure 5:**
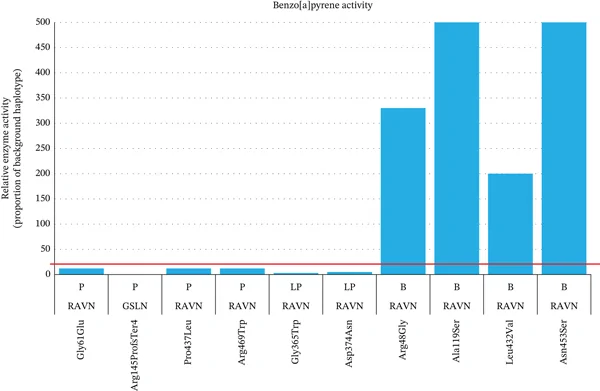
Relative activity against benzol[a]pyrene‐7R‐trans‐7,8‐dihidrodiol of each control variant as a proportion of the background haplotype. Red line represents recommended threshold of 20%, below which PS3_supp can be applied. The variant classification and the background haplotype used is given on the *x*‐axis.

Several variants were measured by multiple studies, often using different methods. Table 4 shows the results for 17*β*‐estradiol activity for control variants measured in at least two different studies for comparison. Although the assay activity agrees between studies for some variants (p.Gly61Glu, p.Arg469Trp, and p.Tyr81Asn), the results were variable between studies for p.Arg117Trp (classified likely pathogenic) and even more so for p.Glu229Lys (classified benign). This phenomenon highlights the highly variable results obtained from different methods. It also underscores the need to interpret cell‐based assay functional results conservatively and to require strong clinical control data to assess the validity of the assay. When a variant has been assessed by two assays meeting the requirements, curators should apply the level of evidence for the assay with the strongest OddsPath (i.e., the best validated) notwithstanding the use of the assay showing the largest effects.

**Table 4:** 17*β*‐estradiol activity measured for control variants by multiple studies. Activity is presented as a percentage of the wildtype activity.

Variant	Protein	Classification	Banerjee et al. [27]	Bagiyeva et al. [35]	Mookherjee et al. [41]	Chavarria‐Soley et al. [29]	Choudhary et al. [36]	Jansson et al. [37]
c.182G>A	p.Gly61Glu	P	0%			11%		30%
c.1405C>T	p.Arg469Trp	P	5%					20%
c.349C>T	p.Arg117Trp	LP	0%	65%				
c.241T>A	p.Tyr81Asn	LB	10%			44%	30%	
c.685G>A	p.Glu229Lys	B	170%		10%	67%	96%	

### 3.3. Comparing Activity Across Multiple Substrates

The study of Banerjee et al. [27] measured both 17*β*‐estradiol and retinol activity for eight different variants. As can be seen in Table 5, the activity of each variant was not always the same with different substrates, even when using the same constructs and cell in the same laboratory. Although all tested variants, except the benign control p.Glu229Lys, had low activity against 17*β*‐estradiol, indicating loss of function, several had very high activity against retinol, suggesting each variant had disparate effects on each substrate. The implications of elevated retinol metabolism on PCG development are not known. There were insufficient LB/B controls assessed for either substrate to apply in a clinical setting; however, in this small study pathogenicity did appear to correlate better with 17*β*‐estradiol activity than with retinol activity.

**Table 5:** Comparison of enzymatic activity against different substrates in Banerjee et al. [27]. Activity is given as a percentage of the RAVN wildtype allele.

Variant	Protein	Classification	Retinol activity	Estradiol activity
c.182G>A	p.Gly61Glu	P	0%	0%
c.1159G>A	p.Glu387Lys	P	0%	0%
c.1331G>A	p.Arg444Gln	P	275%	25%
c.1405C>T	p.Arg469Trp	P	125%	5%
c.1568G>C	p.Arg523Thr	P	210%	10%
c.349C>T	p.Arg117Trp	LP	0%	0%
c.241T>A	p.Tyr81Asn	LB	0%	10%
c.685G>A	p.Glu229Lys	B	140%	170%

## 4. Discussion

Functional data are a powerful contributor to variant classification [43]. However, care must be taken not to overinterpret data from artificial in vitro experiments, particularly when the protein of interest is being overexpressed. The additional guidance by Brnich et al. [34] on the application of functional criteria BS3 and PS3 in the ACMG/AMP guidelines [33] is helpful in ensuring that high‐quality data are applied when appropriate, but that sufficient evidence is available to support the validity of the assay. When considering the available data for variants in *CYP1B1*, we found that only two published assays met the required standards: appropriate technical controls, a sufficient number of benign and pathogenic clinical controls and a clear distinction between benign and pathogenic variants. Both assays expressed the CYP1B1 protein in yeast microsomes on the background haplotype on which it was observed in the patient and compared it with a wildtype construct with the same haplotype. One then measured 17*β*‐estradiol activity with a luminescence‐based read‐out [29, 30], whereas the other used HPLC techniques to measure activity against benzo[a]pyrene [39].

Assays measuring protein abundance [22, 28, 38] and stability [22, 27, 28, 38] as well as activity against EROD [22, 28, 38] were found to have tested appropriate technical and clinical controls when multiple studies were combined; however, they were unable to clearly distinguish between benign and pathogenic variants with overlap in the read‐out for some variants. These assays only tested three likely benign or benign variants (p.Tyr81Asn, p.Glu229Lys, and p.Ala443Gly). Variant p.Tyr81Asn appears to have lower enzymatic activity and reduced stability compared with wildtype but with the small number of controls it is unclear if this is typical of likely benign variants or if p.Tyr81Asn is an outlier. The assessment of additional clinical controls, particularly benign and likely benign variants, could help with the interpretation of these assays in determining an appropriate threshold for indicating pathogenic effects.

Only studies that used the observed background for each variant passed the other criteria for the application of PS3, potentially because they inherently account for differences in background, creating clearer differences between pathogenic and benign variants. All the assays for which we could not determine a consistent threshold tested all variants on the RAVN haplotype. The variability in results may be partly attributed to differential effects of variants on the RAVN haplotype, and most have not been assessed on alternative backgrounds. Ideally, all variants would be tested on all possible backgrounds, and the classification of the variant would be dependent on its genetic context. However, this is unlikely to be feasible in most situations and testing on the background on which it has been observed is a manageable compromise. As the field moves towards multiplexed assays of variant effect (MAVEs), consideration of the haplotype issue for CYP1B1 will be critical, to ensure critical haplotype specific information is not lost in the attempt to use a high throughput assay. Another important consideration is cis versus trans effects. In the context of recessive disease, the activity of the other allele is crucial in determining the eventual phenotype outcome. The effects of different background haplotypes with different baseline activities in combination in an individual have not yet been assessed. There is still much to understand regarding the haplotype specific effects on CYP1B1 activity and its role in causing PCG.

It is interesting to note that the two assays that clearly distinguished pathogenic from benign variants were both conducted in yeast cells. This model system may provide a simpler background platform with fewer confounding factors than mammalian cells with endogenous CYP1B1 expression. Multiple assays also expressed human CYP1B1 in *E. coli*, but all these studies lacked either replicates or negative controls in addition to not meeting a suitable OddsPath. HEK293 cells were the most used mammalian cell type. Although using mammalian cells may more closely replicate human biology, only one study used a cell line derived from ocular tissue, a retinal pigment epithelial cell line RPE8319 [41], and there is no clear link between this cell type and PCG etiology. Future studies may benefit from developing assays that more directly assess the impact of CYP1B1 function on relevant ocular cell types. At present, using nonmammalian model systems and nonocular tissue types to assay enzyme activity and other protein characteristics appears to give consistent results appropriate for use in variant classification for this gene.

The Glaucoma VCEP does not recommend any specific assay or class of assay for the application of BS3, consistent with the advice of Brnich et al. [34], because the biological mechanisms of CYP1B1 glaucoma are not fully determined. With enough controls and well‐validated assays, we will eventually be able to conclude which substrate activities correlate best with variant pathogenicity. We also do not recommend capping the strength of evidence that can be applied. Currently, sufficient controls for these two assays have been tested to allow PS3 to be applied at the supporting level, but this could be increased if additional control data becomes available. Finally, the VCEP does recommend that assays should consider the variant of interest on the background haplotype in which it was observed, as evidence suggests different haplotypes may have different activities. This is particularly important for variants that may have only partial loss of activity. The pathogenicity of such alleles may depend both on the haplotype on which it is observed and the variant on the other allele.

Of the 56 variants with functional data available, only 10 received PS3_supporting in their final classification (Table S1). None changed classification as a result of the functional data. This highlights the current inadequacy of functional data available for this gene. The field should look to assess more variants with the assays highlighted in this review and ideally test more control variants to increase the OddsPath and allow application of PS3 at higher strength. In addition, future assays assessing enzymatic activity need to carefully consider the effects of the background haplotype and assess sufficient control variants to adequately assess the effectiveness of the assay. Protein stability is also a key mechanism where higher quality assays could significantly improve variant classification. The advantage of this class of assay is that it is agnostic to the substrate, but conversely, enzymatic activity still needs to be assessed in stable proteins.

## 5. Conclusion

In summary, the Glaucoma VCEP reviewed 18 reports, of which three covering two assays were found to discriminate between pathogenic and benign variants with sufficient degree of validation. The assay reported by Chavarria‐Soley et al. [29] and Pasutto et al. [30] tested 17*β*‐estradiol activity of the protein expressed in yeast microsomes using a commercially available luminescence kit. The assay reported by Mammen et al. [39] evaluated benzo[a]pyrene activity using HPLC detection, also following expression in yeast microsomes. Consensus review of other assays recommends the use of additional clinical controls to either establish a significant OddsPath or provide greater clarity on appropriate thresholds for assigning pathogenic evidence. The currently available functional data do not contribute significantly to variant classification for *CYP1B1*.

## Funding

This study was funded by a National Health and Medical Research Council (NHMRC) of Australia Investigator grant to E.S. (GNT2016545) and Program Grant to D.A.M. (GNT1150144); Stan Perron Charitable Foundation fellowship to D.A.M.; a Core Grant for Vision Research from the National Eye Institute/National Institutes of Health to the University of Wisconsin‐Madison (P30EY016665); an Unrestricted Grant from Research to Prevent Blindness, Inc. to the UW‐Madison Department of Ophthalmology and Visual Sciences to T.L.Y., S.W.T., and K.W.; and a NIH/NEI grant (R21 EY034251) to S.W.T. Open access publishing was facilitated by Flinders University, as part of the Wiley–Flinders University agreement via the Council of Australasian University Librarians.

## Conflicts of Interest

The authors declare no conflicts of interest.

## Supporting information


**Supporting Information** Additional supporting information can be found online in the Supporting Information section. Table S1: All variants in *CYP1B1* with functional data available in the literature. Figure S1: Article selection process. Figure S2: Functional data from Pasutto et al. [30].

## Data Availability

The data that support the findings of this study are openly available in ClinGen Evidence Repository (ERepo) at https://erepo.genome.network/evrepo/.
