# Supplementary material for: Determining the Quality of Functional Evidence for Coding Variants in CYP1B1 for Use in Variant Classification
Source: Hum Mutat. 2026 Nov 8;2026:6767909. doi: 10.1155/humu/6767909 (PMC13745797; doi:10.1155/humu/6767909)
Supplement: Supplementary file 1 [file HUMU-2026-6767909-s001.docx]

**Supplementary Table 1**: All variants in *CYP1B1* with functional data available in the literature. Variants in bold are control variants (further detail in Table 2). The level at which PS3 was applied to variants tested with assays meeting the criteria is shown, along with the effect on final variant classification.

| **Variant** | **References** | **Level PS3 applied** | **Change in classification?** |
| --- | --- | --- | --- |
| c.74T>G (p.Leu24Arg) | [1] |  |  |
| c.83C>G (p.Ser28Trp) | [2, 3] |  |  |
| **c.142C>G (p.Arg48Gly)** | [4, 5] |  |  |
| c.155C>T (p.Pro52Leu) | [2, 3, 6-8] | Supporting | No (VUS) |
| c.171G>C (p.Trp57Cys) | [2] |  |  |
| **c.182G>A (p.Gly61Glu)** | [2, 3, 5, 6, 9, 10] | Supporting | No (P) |
| **c.241T>A (p.Tyr81Asn)** | [2, 3, 6, 9, 11] |  |  |
| c.266T>C (p.Leu89Pro) | [12] |  |  |
| **c.317C>A (p.Ala106Asp)** | [12] |  |  |
| **c.349C>T (p.Arg117Trp)** | [2, 13] |  |  |
| **c.350G>C (p.Arg117Pro)** | [2] |  |  |
| **c.355G>T (p.Ala119Ser)** | [4, 5] |  |  |
| c.367T>C (p.Phe123Leu) | [12] |  |  |
| c.395T>G (p.Met132Arg) | [2] |  |  |
| c.431A>G (p.Gln144Arg) | [2] |  |  |
| c.432G>T (p.Gln144His) | [2] |  |  |
| c.433C>T (p.Arg145Trp) | [3] |  |  |
| **c.434_443del (p.Arg145ProfsTer4)** | [5] | Supporting | No (P) |
| c.503G>A (p.Gly168Asp) | [3, 8] |  |  |
| **c.517G>T (p.Glu173Ter)** | [6] |  |  |
| c.565G>C (p.Ala189Pro) | [3] |  |  |
| c.570C>A (p.Phe190Leu) | [1] |  |  |
| c.608A>G (p.Asn203Ser) | [9] | Supporting | No (VUS) |
| **c.685G>A (p.Glu229Lys)** | [2, 3, 6, 7, 9, 11, 14] |  |  |
| **c.710C>A (p.Ala237Glu)** | [12] |  |  |
| c.783C>A (p.Phe261Leu) | [2, 6] |  |  |
| **c.784G>T (p.Glu262Ter)** | [6] |  |  |
| c.835C>G (p.His279Asp) | [1] |  |  |
| c.872A>G (p.Asp291Gly) | [2] |  |  |
| c.875T>A (p.Met292Lys) | [2] |  |  |
| c.985G>A (p.Gly329Ser) | [2, 7] |  |  |
| c.986G>T (p.Gly329Val) | [13] |  |  |
| c.986G>A (p.Gly329Asp) | [1] |  |  |
| **c.988_989delinsTT (p.Ala330Phe)** | [11] |  |  |
| c.988G>T (p.Ala330Ser) | [3] |  |  |
| **c.1033_1035del (p.Leu345del)** | [9] | Supporting | No (LP) |
| **c.1064_1076del (p.Arg355Hisfs*69)** | [7] |  |  |
| **c.1093G>T (p.Gly365Trp)** | [5] | Supporting | No (LP) |
| c.1103G>A (p.Arg368His) | [2, 8, 11, 14] | Supporting | No (VUS) |
| **c.1120G>A (p.Asp374Asn)** | [5] | Supporting | No (LP) |
| **c.1159G>A (p.Glu387Lys)** | [2, 15] |  |  |
| **c.1168C>A (p.Arg390Ser)** | [12] |  |  |
| **c.1198C>T (p.Pro400Ser)** | [6] |  |  |
| c.1225G>T (p.Val409Phe) | [2, 3] |  |  |
| **c.1294C>G (p.Leu432Val)** | [4, 5, 7, 16] |  |  |
| **c.1310C>T (p.Pro437Leu)** | [5, 12] | Supporting | No (P) |
| **c.1328G>C (p.Ala443Gly)** | [3, 8] |  |  |
| **c.1331G>A (p.Arg444Gln)** | [2] |  |  |
| c.1334T>G (p.Phe445Cys) | [2] |  |  |
| **c.1358A>G (p.Asn453Ser)** | [4, 5, 16] |  |  |
| c.1394T>C (p.Val465Ala) | [8] |  |  |
| **c.1405C>T (p.Arg469Trp)** | [2, 5, 6, 10] | Supporting | No (P) |
| **c.1409G>A (p.Cys470Tyr)** | [12] |  |  |
| **c.1454C>T (p.Ser485Phe)** | [12] |  |  |
| **c.1568G>C (p.Arg523Thr)** | [2, 14] |  |  |
| c.1589A>G (p.Asp530Gly) | [2] |  |  |

**References**

1.	Faiq MA, Singh HN, Ali M, Dada R, Chan KC, Dada T, et al. Functional genomics of primary congenital glaucoma by pathway analysis and functional characterization of CYP1B1 mutations. Vision Res. 2025;227:108534 doi:10.1016/j.visres.2024.108534

2.	Banerjee A, Chakraborty S, Chakraborty A, Chakrabarti S, Ray K. Functional and Structural Analyses of CYP1B1 Variants Linked to Congenital and Adult-Onset Glaucoma to Investigate the Molecular Basis of These Diseases. PLoS One. 2016;11(5):e0156252 doi:10.1371/journal.pone.0156252

3.	Lopez-Garrido MP, Blanco-Marchite C, Sanchez-Sanchez F, Lopez-Sanchez E, Chaques-Alepuz V, Campos-Mollo E, et al. Functional analysis of CYP1B1 mutations and association of heterozygous hypomorphic alleles with primary open-angle glaucoma. Clin Genet. 2010;77(1):70–8 doi:10.1111/j.1399-0004.2009.01284.x

4.	Hanna IH, Dawling S, Roodi N, Guengerich FP, Parl FF. Cytochrome P450 1B1 (CYP1B1) pharmacogenetics: association of polymorphisms with functional differences in estrogen hydroxylation activity. Cancer Res. 2000;60(13):3440–4

5.	Mammen JS, Pittman GS, Li Y, Abou-Zahr F, Bejjani BA, Bell DA, et al. Single amino acid mutations, but not common polymorphisms, decrease the activity of CYP1B1 against (-)benzo[a]pyrene-7R-trans-7,8-dihydrodiol. Carcinogenesis. 2003;24(7):1247–55 doi:10.1093/carcin/bgg088

6.	Campos-Mollo E, Lopez-Garrido MP, Blanco-Marchite C, Garcia-Feijoo J, Peralta J, Belmonte-Martinez J, et al. CYP1B1 mutations in Spanish patients with primary congenital glaucoma: phenotypic and functional variability. Mol Vis. 2009;15:417–31

7.	Jeannot E, Poussin K, Chiche L, Bacq Y, Sturm N, Scoazec JY, et al. Association of CYP1B1 germ line mutations with hepatocyte nuclear factor 1alpha-mutated hepatocellular adenoma. Cancer Res. 2007;67(6):2611–6 doi:10.1158/0008-5472.CAN-06-3947

8.	Pasutto F, Chavarria-Soley G, Mardin CY, Michels-Rautenstrauss K, Ingelman-Sundberg M, Fernandez-Martinez L, et al. Heterozygous loss-of-function variants in CYP1B1 predispose to primary open-angle glaucoma. Invest Ophthalmol Vis Sci. 2010;51(1):249–54 doi:10.1167/iovs.09-3880

9.	Chavarria-Soley G, Sticht H, Aklillu E, Ingelman-Sundberg M, Pasutto F, Reis A, et al. Mutations in CYP1B1 cause primary congenital glaucoma by reduction of either activity or abundance of the enzyme. Hum Mutat. 2008;29(9):1147–53 doi:10.1002/humu.20786

10.	Jansson I, Stoilov I, Sarfarazi M, Schenkman JB. Effect of two mutations of human CYP1B1, G61E and R469W, on stability and endogenous steroid substrate metabolism. Pharmacogenetics. 2001;11(9):793–801 doi:10.1097/00008571-200112000-00007

11.	Choudhary D, Jansson I, Sarfarazi M, Schenkman JB. Characterization of the biochemical and structural phenotypes of four CYP1B1 mutations observed in individuals with primary congenital glaucoma. Pharmacogenet Genomics. 2008;18(8):665–76 doi:10.1097/FPC.0b013e3282ff5a36

12.	Medina-Trillo C, Ferre-Fernandez JJ, Aroca-Aguilar JD, Bonet-Fernandez JM, Escribano J. Functional characterization of eight rare missense CYP1B1 variants involved in congenital glaucoma and their association with null genotypes. Acta Ophthalmol. 2016;94(7):e555–e60 doi:10.1111/aos.13017

13.	Bagiyeva S, Marfany G, Gonzalez-Angulo O, Gonzalez-Duarte R. Mutational screening of CYP1B1 in Turkish PCG families and functional analyses of newly detected mutations. Mol Vis. 2007;13:1458–68

14.	Mookherjee S, Acharya M, Banerjee D, Bhattacharjee A, Ray K. Molecular basis for involvement of CYP1B1 in MYOC upregulation and its potential implication in glaucoma pathogenesis. PLoS One. 2012;7(9):e45077 doi:10.1371/journal.pone.0045077

15.	Lopez-Garrido MP, Medina-Trillo C, Morales-Fernandez L, Garcia-Feijoo J, Martinez-de-la-Casa JM, Garcia-Anton M, et al. Null CYP1B1 genotypes in primary congenital and nondominant juvenile glaucoma. Ophthalmology. 2013;120(4):716–23 doi:10.1016/j.ophtha.2012.09.016

16.	Aklillu E, Oscarson M, Hidestrand M, Leidvik B, Otter C, Ingelman-Sundberg M. Functional analysis of six different polymorphic CYP1B1 enzyme variants found in an Ethiopian population. Mol Pharmacol. 2002;61(3):586–94 doi:10.1124/mol.61.3.586

**Supplementary Figure 1:** Article selection process

**Supplementary Figure 2**: Functional data from Pasutto et al showing enzymatic activity of each tested variant (panels b,d,f) as well as the background wildtype haplotypes (a,c,e) not included in the original paper.

Molar enzymatic activity, relative CYP1B1 abundance and relative CYP1B1 activity.

**a) & b)** Molar enzymatic activity of haplotypes and mutations.

**c) & d)** Relative CYP1B1 abundance.

**e) & f)** Relative CYP1B1activity.

Different column shades correspond to the different	background	haplotypes. Statistical comparison	was	performed with a one-way ANOVA, followed by the LSD test. The mutants were compared against the corresponding background haplotype. *Significant at the 5% level.
